# Dynamics of soil microbial communities following vegetation succession in a karst mountain ecosystem, Southwest China

**DOI:** 10.1038/s41598-018-36886-z

**Published:** 2019-02-15

**Authors:** Chang Zhao, Jian Long, Hongkai Liao, Chunli Zheng, Juan Li, Lingfei Liu, Mingjiang Zhang

**Affiliations:** 1 Guizhou Key Laboratory of Mountain Environment, Guizhou Normal University, Guiyang, 550001 China; 2 Key Laboratory of Urban Environment and Health, Institute of Urban Environment, Chinese Academy of Sciences, Xiamen, 361021 China; 3 University of Chinese Academy of Sciences, Beijing, 100049 China; 4 Department of Geography and Environmental Science, Guizhou Normal University, Guiyang, 550001 China

## Abstract

The interaction between soil property and soil microbial community in karst area still remains an open question. The characteristics of soil physicochemical properties and microbial community structure and their relationship under five vegetation succession stages (grassland, shrub land, secondary forest, plantation forest, and natural forest) at two soil depths (0–10 cm and 10–20 cm) were explored in a karst mountain ecosystem. We found that soil moisture content (SMC) and pH increased with soil depth across vegetation succession. The highest content of soil nutrients was found in the natural forest stage at both soil depths. The total PLFAs, the abundance of Gram-positive (GP) bacteria, actinomycetes (ACT), fungi, and arbuscular mycorrhizal fungi (AMF) were significantly (P < 0.05) related to variations with soil total carbon (TC) and total nitrogen (TN). Furthermore, the distribution of soil microbial community distinctly differed in vegetation succession both at two soil layers which was demonstrated by Principal-coordinates analysis. Redundancy analyses patterns indicated that soil TC and TN were positively related to cy19:0 and 10Me 16:0, but an opposite relationship with a15:0. Changes of soil microbial communities were significantly determined by vegetation succession, and soil microbial community structure can be a sensitive indicator to reflect the stabilization of karst mountain ecosystem, southwest of China.

## Introduction

Soil microorganisms play critical roles in soil organic matter decomposition, nutrient recycling and ecosystem stabilization, which are major active components of terrestrial ecosystem^1^. Soil microbial biomass, community structure, and physiological activities are sensitive to aboveground vegetation and belowground conditions^2,3^. The production and the composition of vegetation can choose microbial communities of different decomposition types. The organic carbon and nutrient that released from litter decomposition are utilized by the different community of soil microbes^4^. In addition, variations in soil condition including soil moisture, texture and pH also influence the soil microbial community. A clear understanding of the interactions of soil microbial communities and physicochemical properties under different vegetation types will benefit to understand the stability and resilience of the terrestrial ecosystem. The effects of composition and diversity of vegetation on soil microbial properties have been well studied^5^. For example, a significant and linear increased relationship of vegetation diversity with soil microbial catabolic activity, diversity and richness was observed in temperate grass ecosystem^6^. In mixed forest stands, the higher soil microbial biomass and activity than in pure single-species stands is due to the variety of litter and rhizodeposition^7^. Along the vegetation succession stages soil microbial properties (microbial biomass and phylogenetic diversity) increased significantly in karst ecosystem^4^. Such interactions between vegetation and soil microbes are reflected by the fact that changes in microbial composition, activity, and biomass largely resulted from the higher litter quality of vegetation associated with great diversity. For instance, a higher abundance of fungal phospholipid fatty acids (PLFAs) was found in pine forest soils than other vegetation types (beech, oak, spruce)^8^. However, other studies documented that the soil microbial community shows no response to the diversity and the productivity of vegetation^9^. In a late successional stage, the factor is soil water content that impacts the composition of fungal community compared with a diversity of vegetation^10^. Lin, *et al*.^11^ found that soil pH and high soil moisture may reduce the microbial diversity. Higher soil microbial biomass and basal respiration are found in upper soil layer with the forest succession and microbial community shifts toward fungal dominated^12^. Microbial biomass decreases with soil depth likely linked to the decreased organic matter availability in deeper soil layer^13^. In pasture and secondary natural succession, the determine factors of the biomass and catabolic diversity of soil microbial communities are soil organic matter and nitrogen contents^14^. Bacterial and fungi may not mineralize the same types of carbon substrates in the soil, and therefore, shifts in carbon pools may have different impacts on these two groups of microbes.

Recently, the properties of the soil microbial community have been evaluated by community-level physiological profiling (CLPP), PLFA profiles and 16S/18S rRNA genes methods^15^. PLFA analysis is a method that can be useful to estimate the microbial biomass and indicate the microbial physiological status. Identify of signature PLFA biomarkers of prokaryotic and eukaryotic taxa provide an outline of community structure, such as the saturates (17:0, 18:0), monounsaturates (16:1ω7c, 18:1ω7c), mid-chain branched (10Me 16:0)^16^. A further property of this method is that information on the overall metabolic status and stress of a community is also recovered^17,18^. In this study, we applied the PLFA profiles to analyse the properties of soil microbial community in subtropical karst forest ecosystem in Maolan National Nature Reserve, southwest of China. Currently, studies have focused on the changes in soil physicochemical properties and microbial biomass properties during vegetation succession in the fragile karst ecosystem^19,20^. However, little information is available on interactions of the microbial community and physicochemical properties at different soil depths during vegetation succession in the karst ecosystem. Karst landscape is formed from carbonate minerals and the soils are shallow, easily erosive and degenerative. The unique location and topography of the National Nature Reserve provide a succession as following vegetation transect sequence of grassland (GL), shrubs (SL), secondary forest (SF), natural forest (NF), and plantation forest (PF). Which provide a unique site for investigating the changes in soil physicochemical properties and microbial community structure. The objective was to address the following questions, (1) Following the vegetation succession, what is the change of soil physicochemical properties, microbial biomass, and microbial communities differed in different soil depths?; (2) what are the characteristics of soil microbial community in vulnerable karst areas? and (3) what are the key factors that affect the microbial community?

## Results

### Soil physicochemical properties

Soil physicochemical properties were significantly higher than other vegetation types at both depths in the natural forest (P < 0.05, Table 1). The contents of soil TC, TN, and SMC increased significantly at 0–10 cm soil depth with vegetation succession. Soil TC content was 3.91, 2.75, and 1.93 times higher in the natural forest than in grassland, shrub land, and plantation forest, respectively. Similar changes, in the range of 4–1.56 times were found for the TN contents, and SMC increased 0.63–0.24 times for natural forest. At the depth of 10–20 cm, soil TC and TN contents in grassland, shrub land, secondary forest, and plantation forest were comparable, but significantly (P < 0.05) lower than those in the natural forest (Table 1). The means of soil C/N ratios were similar in natural forest and grassland at two soil depths, but significant higher (P < 0.05) than for shrub land. The concentration of TP was the lowest in secondary forest soil than that in shrub land, plantation forest, grassland, and natural forest at both depths (P < 0.05, Table 1). Soil pH increased from grassland and shrub land to secondary forest and plantation forest, with natural forest exhibiting the highest value (Table 1) at both soil depths.

**Table 1:** Soil physicochemical properties under five vegetation successional stages at 0–10 cm and 10–20 cm depths.

Stage	Depth (cm)	Total C (%)	Total N (%)	C/N ratio	Total P (mg/g)	pH	SMC (%)
GL	0–10	2.18 ± 0.21 a	0.23 ± 0.02 a	9.66 ± 0.19 c	0.85 ± 0.01 c	6.04 ± 0.03 ab	23.8% ± 0.74 a
	10–20	1.68 ± 0.04 a	0.18 ± 0.01 a	9.12 ± 0.26 d	0.86 ± 0.11 c	6.22 ± 0.09 b	19.7% ± 0.69 a
SL	0–10	2.85 ± 0.27 ab	0.45 ± 0.03 b	6.35 ± 0.20 a	0.45 ± 0.04 ab	5.75 ± 0.40 a	26.3% ± 0.32 b
	10–20	2.16 ± 0.03 a	0.38 ± 0.01 a	5.69 ± 0.03 a	0.41 ± 0.07 a	5.44 ± 0.56 a	24.2% ± 0.86 c
SF	0–10	2.76 ± 0.19 ab	0.30 ± 0.02 ab	9.29 ± 0.10 bc	0.41 ± 0.01 a	6.27 ± 0.16 bc	27.3% ± 0.58 c
	10–20	1.56 ± 0.16 a	0.21 ± 0.02 a	7.42 ± 0.69 b	0.31 ± 0.03 a	6.82 ± 0.22 b	20.6% ± 0.16 b
PF	0–10	3.65 ± 0.41 b	0.45 ± 0.07 b	8.09 ± 0.53 b	0.74 ± 0.09 bc	6.53 ± 0.01 c	31.1% ± 0.27 d
	10–20	2.03 ± 0.22 a	0.24 ± 0.02 a	8.46 ± 0.28 c	0.64 ± 0.05 b	6.29 ± 0.07 b	25.6% ± 0.51 c
NF	0–10	10.7 ± 0.67 c	1.15 ± 0.22 c	9.46 ± 1.44 c	1.70 ± 0.37 d	7.28 ± 0.04 d	38.7% ± 0.44 e
	10–20	7.74 ± 1.94 b	0.83 ± 0.24 b	9.37 ± 0.53 d	2.20 ± 0.07 d	7.46 ± 0.02 c	31.8% ± 0.86 d

GL, grassland; SL, shrub land; SF, secondary forest; PF, planted forest; NF, natural forest; SMC, soil moisture content. Data are means ± SD (*n* = 3). Different litters indicate significant differences for each column in the same soil depth among vegetation types (*P* < 0.05).

### Soil microbial community structure

**Figure 1:**
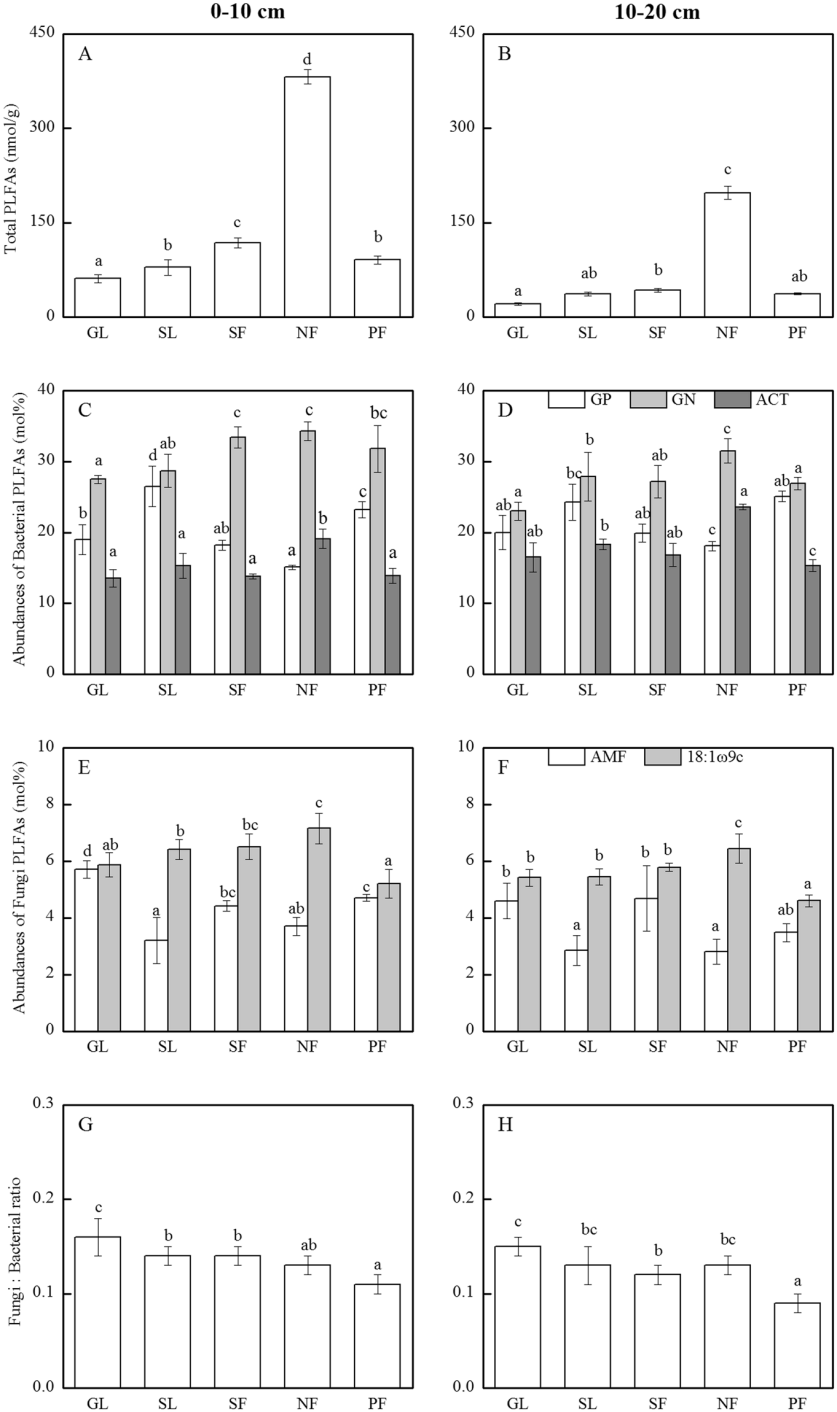
The compositions of soil microbial community at each vegetation successional stages in 0–10 cm and 10–20 cm depths in karst ecosystem. GL: grassland; SL: shrub land; SF: secondary forest; PF: planted forest; NF: natural forest; GP, Gram-positive bacteria; GN, Gram-negative bacteria; ACT, actinomycetes; AMF, arbuscular mycorrhizal fungi; F/B ratio, the fungi to bacterial ratio. (**A**) and (**B**) Representation of total PLFAs in 0–10 cm and 10–20 cm depths, respectively. (**C**) and (**D**) Representation of abundance of GP, GN and ACT in 0–10 cm and 10–20 cm depths, respectively. (**E**) and (**F**) Representation of abundance of AMF and 18: 1ω9c in 0–10 cm and 10–20 cm depths, respectively. (**G**) and (**H**) Representation of Fungi:Bacterial ratio. For the same parameter, histograms show the mean with SD bar, n = 3. Different litters indicate significant differences in the same soil depth (*P* < 0.05).

The highest PLFAs was found in natural forest soil among all vegetation stages at both soil depths (Fig. 1A,B). Soil microbial biomass varied difference with soil depth and vegetation succession except for plantation forest stage compared with shrub land stage. In addition, the abundances of bacterial and fungal communities were also affected by vegetation succession and soil depth. The relative abundances of GP were decreased gradually form grassland stage (19.1% and 20.1%), and secondary forest stage (18.1% and 19.4%) to natural forest stage (15.1% and 17.9%) at both depths, respectively (Fig. 1C,D). In contrast, the abundance of GN has shown an increasing trend in the range of 22.1–34.3% along the vegetation succession was obtained for the abundances of GN at two soil depths. Whilst, the ratio of GP: GN decreased from 0.71 and 0.87 in grassland to 0.44 and 0.54 in the natural forest at 0–10 cm and 10–20 cm depths. The abundances of the ACT were comparable among all vegetation stages except for natural forest at both depths.

The abundances of the two fungal biomarkers AMF (16:1ω5c) and 18:1ω9c were varied with vegetation succession and soil depth (Fig. 1E,F). The values of 18:1ω9c increased in the following order: PF < GL < SL < SF < NF, varied from 5.21% to 7.16% at 0–10 cm depth and 4.61% to 6.45% at 10–20 cm depth, respectively. The abundance of AMF was highest in the grassland stage, lowest in the natural forest stage, and intermediate in the secondary forest and planted forest stages. For the F: B ratio, a decrease along the vegetation succession was obtained, from 0.16 under grassland stage to 0.11 under natural forest stage at 0–10 cm depth (Fig. 1G). However, the F: B ratio at 10–20 cm depth under the natural forest stage was higher than the plantation forest stage and comparable with the shrub land stage (Fig. 1H).

### The relationship between soil physicochemical properties and soil microbial properties

Spearman correlation analysis showed soil physicochemical properties were significantly and positively correlated with the content of total PLFAs and the abundance of the ACT (P < 0.01) except for soil C/N ratio at 0–10 cm depth (Table 2) across all successional stages. However, the abundance of GP was negatively correlated with soil physicochemical property (P < 0.01). The abundances of 18:1ω9c and AMF showed negative correlations with soil TN and TC, respectively. At soil depth of 10–20 cm, there were significant positive relationships between soil TN and the content of total PLFAs, the abundance of ACT, GP, 18:1ω9c, and AMF. Similar relations were found between soil TC and the content of total PLFAs, and the abundance of ACT, GP and 18:1ω9c.

**Table 2:** Spearman correlation analysis between soil microbial compositions and physicochemical properties at 0–10 cm and 10–20 cm depths, respectively.

Stage	Depth	T-PLFAs	GP	GN	ACT	18:1ω9c	AMF	F/B
TN	0–10 cm	0.930**	−0.548*	0.039	0.806**	0.568*	−0.636*	0.038
	10–20 cm	0.895**	−0.528*	0.040	−0.714**	0.707**	−0.536*	0.225
TC	0–10 cm	0.979**	−0.682**	0.137	0.861**	0.568*	−0.612*	0.049
	10–20 cm	0.923**	−0.635*	0.162	−0.700**	0.731**	−0.477	0.165
C/N	0–10 cm	0.349	−0.730**	0.588*	0.236	0.118	0.152	0.091
	10–20 cm	0.414	−0.622*	0.522**	−0.173	0.245	0.236	−0.149
TP	0–10 cm	0.862**	−0.704**	0.197	0.680**	0.359	−0.351	0.089
	10–20 cm	−0.134	0.399	0.441	0.582*	−0.345	0.235	−0.547*
SMC	0–10 cm	0.904**	−0.589*	0.238	0.840**	0.366	−0.556*	−0.227
	10–20 cm	0.186	0.334	0.254	0.346	0.046	−0.240	−0.410
pH	0–10 cm	0.894**	−0.737**	0.515*	0.749**	0.347	−0.281	−0.299
	10–20 cm	−0.072	0.035	0.853*	0.518*	−0.112	0.112	−0.743**

GP, Gram-positive bacteria; GN, Gram-negative bacteria; ACT, actinomycetes; AMF, arbuscular mycorrhizal fungi; B, bacterial. *Indicated the significance at *P* < 0.05; **Indicated the significance at *P* < 0.01; *n* = 3.

**Figure 2:**
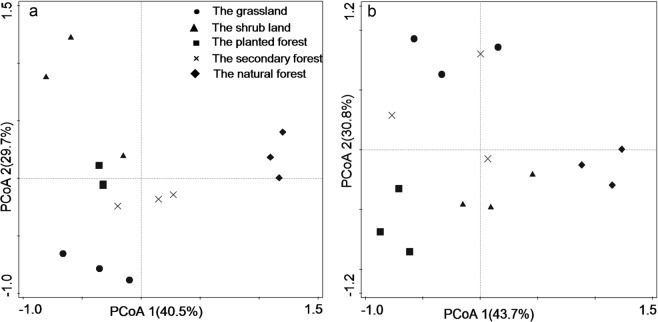
Principal-coordinates analysis (PCoA) of the abundance of soil microbial signature PLFAs based on Bray-Curtis distance matrix show the distribution at 0–10 cm (**a**) and 10–20 cm (**b**) depths among vegetation types.

**Figure 3:**
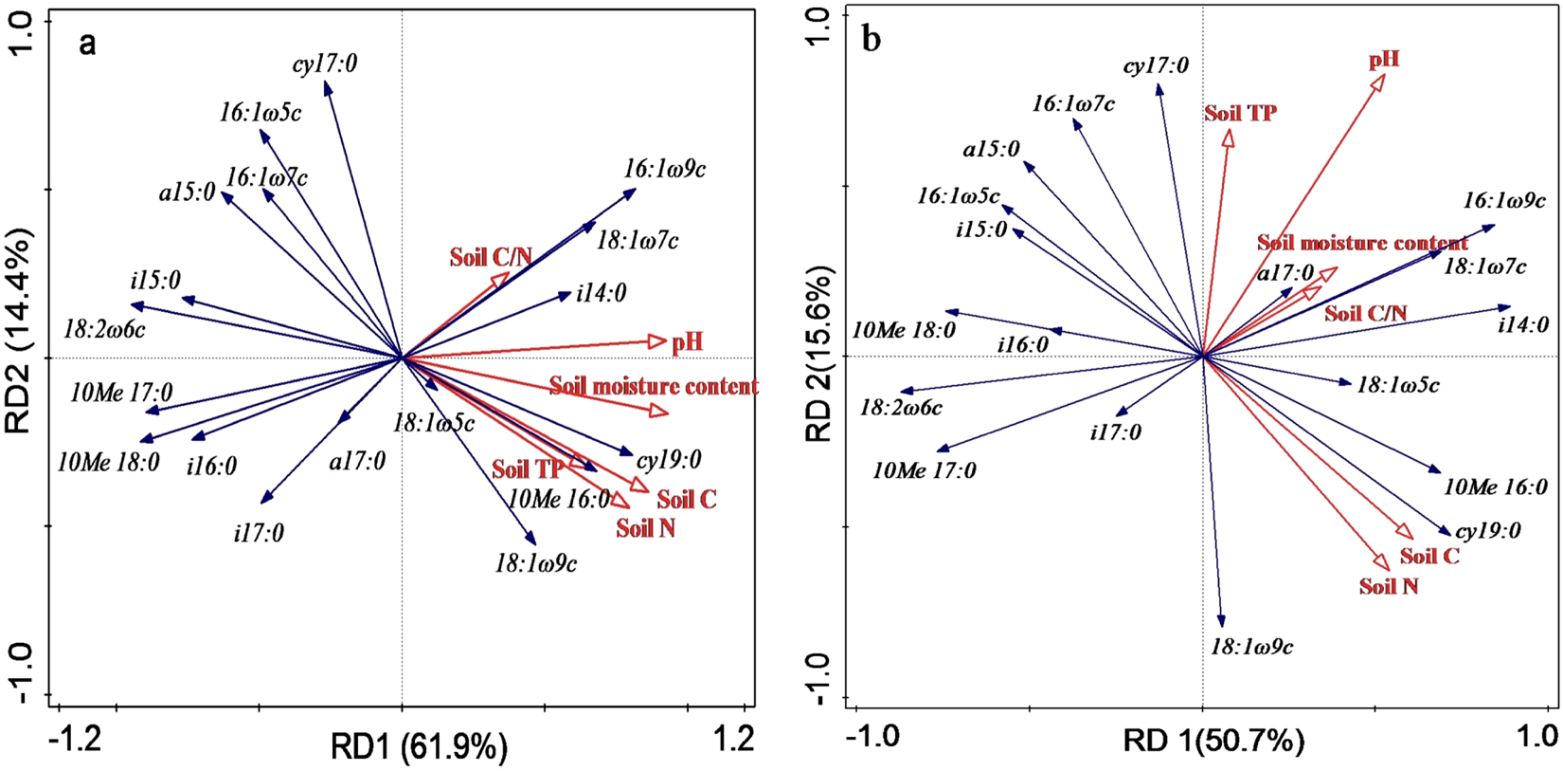
Redundancy analyses for soil microbial biomarkers based on physicochemical properties at 0–10 cm (**a**) and 10–20 cm (**b**) depths under all stages in karst ecosystem.

To further assess the relationship between soil microbial communities and physicochemical properties, PCoA and RDA were performed (Figs 2 and 3). The microbial community structure varied considerably among the five vegetation stages by PCoA analyses (Fig. 2a,b). The components of PCoA1 and PCoA2 accounted for 70.2% of the total variance in soil microbial composition, 40.5% of this variance was explained by PCoA1, and another 29.7% was explained by PCoA2. Along the PCoA1 axis, different vegetation types were well separated (Fig. 2a). At 10–20 cm soil depth, the PCoA1 and PCoA2 accounted for 74.5% of the variation together. Along PCoA1 axis, plantation forest stage is on the left. The PCoA1 might present the degree of land degradations due to anthropogenic interference in karst ecosystem.

RDA showed that soil microbial community structure was related to soil TC, TN, and pH. All of the physicochemical properties explained 76.4% (RD1 61.9% and RD2 14.4%) and 66.3% (RD1 50.7% and RD2 15.6%) of the variance at 0–10 cm and 10–20 cm soil depths, respectively (Fig. 3a,b). Soil TC and TN were mainly related to the ACT PLFA marker (10 Me 16:0) and Gram-negative bacteria PLFA marker (cy19:0), while pH was correlated with the AMF PLFA marker (16:1ω9c) at two soil depths.

## Discussion

In our study, five sampling soil sites are formed by the same carbonate rock and the effect of soil texture on physicochemical properties is eliminated during vegetation succession in karst mountain ecosystem. Large variability has been observed previously^21^ in soil nutrient and microenvironment following vegetation succession. Those variabilities are attributed to the changes in tree species, productivity and soil microclimate. Soil pH value ranged from 5.44 under shrub land stage to 7.46 under natural forest stage in the study (Table 1). The increasing trend in pH was in accordance with the results of the same karst region of Zhang, *et al*.^19^, but an opposite tendency was found in the research of Loess Plateau of Tian, *et al*.^22^. Therefore, this phenomenon was produced due to the changes of microenvironments. The difference of soil pH in southwestern karst and Loess Plateau was controlled by the changes of latitude zonality, the differences of regional climate and the species of vegetation. There have an indirectly plant driven observed between soil pH and microbial community structure. In our study, five sample soils are acidic except natural forest soil. Plant root exudates and organic acids produced during decomposition of the litter were the main factors that caused the soil pH decreased. Our study showed a neutral pH value in natural forest soil. This could be attributed to shifting in the calcium concentration and quality of the litter inputs. In addition, higher calcium concentration decreases soil acids reaction, and affects soil availability nutrient recycling and utilizating.

The contents of soil TC and TN were generally enhanced as successional stages changed from grassland, shrub and secondary forest to natural forest. It confirmed that with vegetation succession, the enhancement of plant productivity and litter biomass would lead to soil organic matter input increased, the decomposition rate decreased^23^ and the soil water holding capacity improved in karst ecosystem (Table 1). This result was consistent with Mackay, *et al*.^24^ who reported that the increase of soil carbon content was correlated with higher plant productivity and soil moisture content due to a rise vegetation density and species in karst forest ecosystem. The average contents of soil TC (9.31%), TN (0.99%), and TP (1.95 g/kg) under natural forest throughout the 0–20 cm depths in our study and were significantly higher than the aspen woodland (8.6%, 0.79%, 0.65 g/kg) in semi-arid loess plateau^25^ and the primary zonal vegetation types (2.98%, 0.21%, 0.24 g/kg) in subtropical zone^26^. This might be attributed to the hot and humid climate conditions and the greater concentration of calcium in karst mountain area, which were conductive to the physical protection of macro-aggregate (>1 mm diameter) and the growth of microorganisms^27,28^. But the average contents of 0–20 cm depth of soil TC, TN, and TP were lower than the results of Wu, *et al*.^29^ reported in the same area. Those differences indicated that the effects of outcrop rock rate, vegetation communities, and microenvironment on soil nutrient were heterogeneity in karst mountain ecosystem. SOC, TN, Alkali-hydrolyzable nitrogen (AN) concentrations showed increases in two soil depths along with vegetation succession, and declined along with soil depth in five vegetation stages^19,30^. Some previous studies reported that SOC, TN contents increase along succession proceed^31,32^. Differences in plant species, composition, and diversity affected the microbial community structure by the changes in soil carbon quality. In addition, the C/N ratio in shrub land stage (6.1) was significantly lower than the other stages (8.9–9.4) and San-do-qing forest successional stages (12.9–14.6)^33^. The results further obtained that soil nutrient was limited by the nitrogen in the karst ecosystem, and the increase of C_3_ plants can increase soil C and N accumulation in forest ecosystem^34^. Therefore, soil physicochemical properties were significantly affected by vegetation succession and may provide a better opportunity to assess the quality of soils and the stabilization of karst ecosystems.

The biomass of soil microbes is significantly higher in natural forest stage than that in secondary forest, shrub land, plantation forest and grassland stages at two soil depths (Fig. 2). This phenomenon may attribute to the enhancement of plant species along the vegetation succession. The organic matter promotes the formation of soil aggregation and the accumulation of soil nutrient, especially in surface soil. Some researchers also confirmed that the influence of litter input on the microbial community was stronger in surface soil than that in deeper soil^35,36^. Hence, the total amount of soil microbial PLFAs is significantly affected by aboveground vegetation types in surface than that in deeper soil at karst area. The abundances of soil microbial PLFAs showed that soil microbial community composition significantly changed with vegetation succession and soil depth. In our study, the declined abundance of GP and the increased abundance of GN are found from grassland stage to natural forest stage at each soil depths, but there was no significant change in the ACT. This phenomenon supports the argument that vegetation succession probably provides more plant materials, which were regarded as the preferential microbial C resource for GN bacteria^37^. More microbial C resource accelerates the propagation of GN bacteria and shifts soil microbial community towards species tending to accumulate soil organic matter. With soil depth increased, the relative abundances of GP and GN showed a comparable decreasing trend but a remarkable increasing was observed by 10.1%-23.6% in ACT (Fig. 1C,D). For the physiology of ACT may have produced enzymes targeting complex and recalcitrant biopolymers in deeper soil where has reserved substantial amounts of recalcitrant carbon^38,39^. A higher abundance of AMF was found in grassland stage as opposed to the natural forest stage. The synergistic effect of grassland plant on soil fungal community was through the composition of plant^40^, essentially was affected by the activity of AMF relevant to plant roots or rhizosphere^41^. The lowest values of fungal biomarker 18:1ω9c and F:B ratio were found in plantation forest soils. 18:1ω9c biomarker, played a dominant role in soil fungal community, and was more sensitive to soil disturbance than AMF. Moreover, the abundance of AMF decreased with the increasing plant diversity from grassland to natural forest stage. This may be related to the ability of AMF degrading the complex lignocellulose component, similar to Antoninka, *et al*.^42^ reported in Cedar Creek ecosystem. In our research, the increased bacterial and the decreased abundance of fungi resulted in the ratio of F:B decline. Wan, *et al*.^43^ found a greater abundance of fungi and a higher F:B ratio in mineral soil under coniferous forest, consistent with the consequence of our research in grassland soil. These results confirmed the predominance function of soil fungi in decomposing recalcitrant substrates^44^ and bacterial populations were more competitive for utilizing available substrates^45^. Taken together, our results demonstrated that vegetation succession and soil depth have important effects on soil microbial community structure in the karst ecosystem. The F:B ratio was particularly sensitive to soil disturbance and was declined when cultivation intensifies and N fertilization input increased^46^.

Across all the vegetation succession stages, the variations of soil microbial community structure associating with soil TC, TN, SMC, and pH (Fig. 3) are explained by the different quality and quantity of litter and the physicochemical conditions of the soil. A significant decrease in the abundance of GP with increasing pH by contrary in GN and ACT also has been observed in terrestrial soil types^47^. An aggregation effect of ACT and 18:19c profiles are associated with the gradually increased soil pH (5.59–7.34, Table 1) from grassland to forest stage. In other investigations, pH ranges in 3.30–7.24^48^ and 3.30–7.37^49^ showed a strong effect on soil bacterial and fungal community structure. Studies in Arctic tundra and alpine grassland soils have reported that competitive interactions between fungi and bacteria lead to opposite relationship with soil pH^50^. Moreover, the correlation between soil pH and microbial community might actually be related to the soil moisture and carbon availability^51^. Previous studies have shown that soil moisture content had a positive correlation with organic carbon across the vegetation succession, which co-varied with soil pH^52^. Both soil C and N contents are improved with vegetation succession from grassland to forest stage, which stimulates microbial metabolisms and changes bacterial and fungal community structure by the increased soil amino sugars^53^. Changes of soil C/N ratio could significantly affect the structure of soil microbial community and the nitrogen availability supply for plants. But no significant relationship between C/N ratio and soil microorganism is found in our study. When soil C/N ratio is below^20^, both fungi and bacterial are often C-limited^54^. Karst environment structure is distinctively fragile and the faster decomposition rate of soil organic carbon limits the development of soil microorganism. In our results, soil C/N ratio ranges of 5.59–9.66 indicate that the growth of soil microorganism is controlled by soil carbon resource across vegetation succession.

## Conclusion

Our results confirm that the total biomass of soil microorganism increased with the process of natural vegetation succession, as well as the concentration of soil TC and TN at top- and subsoils. Soil carbon and nitrogen play an important role in shaping the distribution of soil microbial communities at five vegetation succession stages. GP and ACT are the dominant microbial communities changed by soil physicochemical properties. The ACT biomarker 10Me 16:0 and GN bacteria biomarker cy19:0 are positively related to soil TC and TN. Soil microbial community composition is a sensitive indicator reflecting the stabilization of soil nutrient cycling and the sustainability of the karst forest ecosystem. Further research will focus on exploring the effects of soil physicochemical properties on microbial function diversity in determining the key functional microbial components in karst forest ecosystem.

## Materials and Methods

### Study area and sample collection

This study was conducted in Maolan Karst National Nature Reserve, Guizhou province, Southwest China. This region is the largest primary forest under the same latitude in the global with an area of 221 km^2^ (107°52′~108°05′N, 25°09′~25°20′E) and mean elevation of 550–850 m and has a subtropical humid monsoon climate. The evergreen broad-leaf forest cover is about 87%, with a mean annual temperature of 15 °C and the air relative humidity of 83%. The mean annual precipitation is 1389 mm, of which more than 65% occurs during the time from June to September. Sites for grassland, shrubs, secondary forest, and natural forest have been maintained for over centuries, and the planted forest was converted from grassland since the 1970s. All of the soils are derived from the identical limestone parent materials. More detail land information and dominant plant species in vegetation successional sites were showed in Table 3.

**Table 3:** Characteristics of five successional stage sites in karst ecosystem.

Successional stages	Longitude and latitude	Slop (°)	Tree coverage (%)	Elevation (m)	Dominant species
NF	N25°12′48″E108°1′54″	10–20	93	730	*Distylium chinensis*, *Cyolobalanopsis glauca*, *Platycarya longipes*, *Boniodendron minus*, *Symplocos adenopus*
SF	N25°14′57″E108°0′32″	15–20	85	745	*Toona sinensis, Gleditsia sinensis, Radermachera sinica*
PF	N25°10′34″E108°0′29″	20–25	85	720	*Ampelocalamus salcareus*
SL	N25°13′13″E108°0′51″	20–25	75	708	*Rosa cymosa Tratt, Pyracantha fortuneana and Diospyros dumetorum*
GL	N25°12′53″E108°0′47″	10–15	50	720	*Miscanthus floridulus and Imperata cylindrical*

NF, the natural forest stage; SF, the secondary forest stage; PF, the planted forest stage; SL, the secondary forest stage; GL, the grassland stage.

Soil samples were collected in middle February 2017. Three sampling quadrats were selected (30 m × 30 m) at each of the vegetation successional sites. Five to eight soil samples were collected at depths of 0–10 and 10–20 cm, then mixed as one composite sample at the same depth in each quadrat, six composite samples were taken in each vegetation type at two soil depths. Stones, roots and plant debris were removed from the fresh composite soils. Each sample was divided into three sub-samples. One was air-dried and sieved through 2 mm and 0.15 mm mesh for soil physicochemical analyses, the second part was immediately stored at −80 °C used for determining the PLFAs, and the remaining was stored at 4 °C for further analysis.

### Soil analysis

Soil moisture content (SMC) was measured by drying soil samples at 105 °C for 24 h. Soil pH was determined with a soil: liquid ratio of 1:2.5 using PHSJ-5 acidity detector by the potentiometric method. Total carbon (TC) and total nitrogen (TN) were determined by an Elemental Analyzer (Vario Macro CNHS, Hanau, Germany). Total phosphorus (TP) was analyzed using a UV-spectrophotometer (Varian Cary 100, USA) by molybdenum blue colorimetric method after digestion with sulfuric acid and perchloric acid.

Soil microbial community composition at 0–10 cm and 10–20 cm soil depths were analysed through assessment of PLFAs. PLFAs were extracted from subsamples (1.5 g) using a mixture of chloroform, methanol, and citrate buffer (1:2:0.8 V/V/V) according to a method modified from White, *et al*.^55^. Soil samples were extracted with 15 mL solvent in a shaker for 2 h and repeated once. After 10 min centrifuging, the supernatant was removed and then evaporated under N_2_ to 1 mL. Each sample sequentially eluted with chloroform and acetone by solid phase extraction (SPE) columns and phospholipid fraction was collected in a small test tube with 5 mL of methanol and finally evaporated under N_2_. After the addition of internal standard (nonadecanoic acid methyl ester 19:0) in the small test tubes, the samples were evaporated again under N_2_. For methanolysis, the phospholipid fraction was incubated at 37 °C for 15 min and then neutralised with 0.3 mL acetic acid and 2 mL of ultrapure H_2_O. Twice extractions were carried out with a mixture of 2 mL hexane: chloroform (4:1 V/V) and the organic phases were combined. The organic layer was collected and evaporated again under N_2_. Each sample was resuspended in 200 μL of hexane and analysed using gas chromatography (Agilent 6890 Series, USA). The chromatography was conducted with a BPX 70 column (50 m × 0.32 mm I.D., 0.25 μm film thickness), using He as a carrier gas at a flow rate of 1.3 mL min^−1^. The initial temperature program of 120 °C, ramped to 135 °C at 4 °C min^−1^, then to 230 °C at 20 °C min^−1^, and held 260 °C for 1 min. The concentrations of the individual compounds were obtained by comparing the peaks with a standard mixture of saturated fatty acids and unsaturated fatty acids by combination with the MIDI microbial identification system (MIDI, Inc., Newark, DE).

Total PLFA was calculated as the sum of all phospholipid fatty acids and measured as microbial biomass. All iso and anteiso branch chain fatty acids (i14:0, i15:0, i16:0, i17:0, a15:0, a17:0) as Gram-positive bacteria (GP), monounsaturated and cyclopropane fatty acids (16:1ω9c, 16:1ω7c, 18:1ω7c, 18:1ω5c, cy17:0, cy19:0) were designated, as Gram-negative bacterial (GN)^56^, and the PLFAs 18:1ω9c and 18:2ω6,9c were used as indicators of fungi^57,58^. While 16:1ω5 was designated as arbuscular mycorrhizal fungi (AMF)^59^. The PLFAs 10Me16:0 and 10Me18:0 were used to indicate soil actinomycetes (ACT)^60^. For each sample, a total of 21 PLFAs were included, the abundance of individual PLFAs was calculated in absolute concentration of C (nmol PLFA-C g^−1^ soil), and then converted to mole percentage PLFA-C. Fatty acids percent >4% were included in the community composition analysis. Structure of soil microbial community was measured using the fungi: bacteria ratio (F:B) and the GP: GN bacteria ratio.

### Data analysis

All data processing and statistical analyses were performed with Microsoft Excel 2003 and SPSS 18.0 for Windows. The significant differences in soil physicochemical and microbial properties among the studied vegetation successional stages were tested with one-way analysis of variance (ANOVA) followed by least square differences (LSD) tests at 0.05. The relationship between soil physicochemical properties and microbial composition was analyzed by Spearman correlation tests. Soil PLFAs was compared using a principal coordinate analysis (PCoA) to test the effects of vegetation succession and soil depth on microbial distribution. Redundancy analysis (RDA) was performed to determine which environmental factors were related to the soil microbial community composition.

## Data Availability

All data generated or analyzed during this study are included in this published article.
